# Meta-Omics Tools in the World of Insect-Microorganism Interactions

**DOI:** 10.3390/biology7040050

**Published:** 2018-11-27

**Authors:** Antonino Malacrinò

**Affiliations:** Department of Physics, Chemistry and Biology (IFM), Linköping University, 58183 Linköping, Sweden; antonino.malacrino@liu.se or antonino.malacrino@gmail.com

**Keywords:** metagenomics, metatranscriptomics, metaproteomics, microbiome, insect pests

## Abstract

Microorganisms are able to influence several aspects of insects’ life, and this statement is gaining increasing strength, as research demonstrates it daily. At the same time, new sequencing technologies are now available at a lower cost per base, and bioinformatic procedures are becoming more user-friendly. This is triggering a huge effort in studying the microbial diversity associated to insects, and especially to economically important insect pests. The importance of the microbiome has been widely acknowledged for a wide range of animals, and also for insects this topic is gaining considerable importance. In addition to bacterial-associates, the insect-associated fungal communities are also gaining attention, especially those including plant pathogens. The use of meta-omics tools is not restricted to the description of the microbial world, but it can be also used in bio-surveillance, food safety assessment, or even to bring novelties to the industry. This mini-review aims to give a wide overview of how meta-omics tools are fostering advances in research on insect-microorganism interactions.

## 1. Introduction

It is widely acknowledged that microorganisms are the main drivers of several fundamental physical, chemical and biological phenomena [1]. As scientific techniques and instruments evolve, the role of microorganisms in shaping the lifestyle of other organisms becomes clearer. Indeed, the study of microbial ecology is widely spreading all around the scientific community, starting from human microbiology and expanding within other research topics, from marine ecology to food science and insect science [2,3,4].

Insect-associated microbial communities are attracting increasing interest, mainly because of their ecological and economical importance. Microorganisms have been investigated for the effects on their host partner, by directly mediating interactions with other species, or indirectly by impacting the host genetic diversity, with effects visible at community level [5]. Moreover, microorganisms can help insects to counteract plant defenses, provide protection from natural enemies, influence the reproductive system [5] and help to thrive on nutritionally marginal diets. These are just few examples of the plasticity of insect-microorganisms relationship.

More recently, the study of host-microorganism interactions has been boosted by the introduction of meta-omics techniques. A wide range of research described the insect-associated microbial community using meta-omics tools, spanning several host taxa: *Pentalonia nigronervosa* (Coquerel) [6], *Lutzomyia longipalpis* (Lutz & Neiva) [7], *Lutzomyia intermedia* (Lutz & Neiva) [8], *Rhynchophorus ferrugineus* (Olivier) [9], *Apis mellifera* L. [10,11], *Formica exsecta* Nylander [12] and *Dendroctonus* bark beetles [13] to name a few. This mini-review briefly introduces the techniques and tools currently available and highlights some of the developments these techniques are facilitating in insect science.

## 2. Meta-Omics Techniques: An Overview

Peršoh [14] provides a thorough overview of the development of the term ‘meta-omics’, which currently indicates a defined group of techniques used to characterize communities of organisms: meta-genomics, meta-transcriptomics, meta-proteomics and metabolomics. Metagenomics tools are helpful to identify the pool of genomes in a sample. This term has been used for the first time by Handelsman et al. [15] to indicate the set of microbial genomes in a soil sample. Indeed, the metagenome represents the complex of the genomes of all the organisms that are present in a specific sample. A more targeted version of metagenomics, called metabarcoding [16], aims at the taxonomical reconstruction of biological communities in a specific sample, using a short nucleotide fragment called barcode (e.g., 16S, 18S, ITS, COI) as proxy for identification. Both metagenomic and metabarcoding approaches are useful to qualitatively evaluate the diversity of organisms in a sample, but also to inform on the relative taxonomical abundance and on the presence of specific genes in that sample. However, these approaches just tell us who is in there (taxonomic reconstruction), and what potentially is doing (gene identification). The likely functional role of reconstructed communities can be predicted using tools like PICRUSt [17] for bacteria, or FUNGuild for fungi [18].

A metatranscriptomic approach can also be used for taxonomical reconstruction, provided that we have enough information on transcriptomes, but also tells us which genes the community is expressing in that sample [19]. Therefore, in addition to knowing who is inhabiting our sample, we are able to get information on which biological processes are active. However, changes in gene expression are not always followed by phenotypical responses, so meta-proteomics and metabolomics can help us to understand what the microbial community is actually doing [19,20,21].

## 3. Insect Pests

The intensification of agriculture and the simplification of agroecosystems heightened the damage to crops by pests. To counteract these losses, farmers have applied physical, biological and chemical measures. Synthetic pesticides boosted agricultural production with their striking effects, and the practice quickly became widely used worldwide. Besides the beneficial effects on agriculture, we are fully aware of the health and environmental impact of pesticides [22].

Microorganisms are key actor in pests’ life and interaction with the environment, and their manipulation could mitigate the impact of pests on plant productivity, thus leading to a reduction of chemical inputs. Indeed, microbes are able to affect insects’ fitness, to improve their resistance to stress and to affect gene flow [23,24]. The psyllid *Bactericerca cockerelli* (Sulc) that exploits its symbiont to modulate plant defensive gene expression [25], bark and ambrosia beetles that exploit fungi for dietary needs but also to overcome plant defenses [26], and the maintenance of leaf green islands by symbiotic bacteria which is fundamental for leaf miners’ survival [27] are just few examples of the plasticity of these interactions [28].

Meta-omics techniques revealed useful information in this field. Sequencing of diamondback moth (*Plutella xylostella* L.) metagenome, together with the functional profile of its microbiota, revealed a major role of bacterial taxa in the adaptation to detoxify plant defense compounds [29]. Saha et al. [30] used metagenomics to study the endosymbiont diversity of *Diaphorina citri* Kuwayama, vector of Candidatus *Liberibacter asiaticus* know causal agent of citrus greening disease. A metatranscriptomic approach has been used by Cox-Foster et al. [31] to investigate the causal agent of colony collapse disorder in honeybees, suggesting a correlation between the disease and the detection of Israeli acute paralysis virus. A similar approach allowed for the identification of candidate pathogens responsible for the decline of the invasive yellow crazy ant (*Anoplolepis gracilipes* Smith) in Australia, providing pivotal information that can be developed into pest management programs [32].

The use of meta-omics techniques to unveil the diversity of viruses spreading different ecosystems is providing more insights into the ecological importance of viruses [33,34,35] and can serve as basis to enhance future biocontrol programs. For example, a metagenomics approach has been used to study the viral community of different species of mosquitoes, revealing a very diverse community of animal, plant, insect and bacterial viruses [36].

## 4. Gut Microbes

Microorganisms are able to play a fundamental role in insects’ nutrition, sometimes in a symbiotic way, increasing the availability several nutrients or regulating their allocation [37]. Evolution led insects to exploit microorganisms in order to adapt to nutrient-limited environments, feeding directly on them or relying on them to pre-digest refractory diets [38]. These microbial symbionts could be vertically transferred from one generation to the next, horizontally among individuals of the same species, but also can be acquired directly from the environment [39,40]. Sometimes, this association is so strict that experimental replacement of gut microbial communities in insect herbivores can lead to a decrease in their performance [39]. An important role of gut microbial community is performed by detoxifying plant secondary metabolites and by helping their host to better exploit their diet [41]. The analysis of the gut microbiota of *Hyles euphorbiae* (L.) and *Brithys crini* (F.), for example, revealed a community dominated by *Enterococcus*, with a likely role in helping these insects to feed on toxic plants [42].

More recent studies highlighted the effects of host plant species on the microbiota associated with polyphagous species. This has been observed to occur, for example, in *Acyrthosiphon pisum* Harris [43], *Helicoverpa armigera* (Hübner) [44], *Phylloxera notabilis* Pergande [23], *Melitaea cinxia* (L.) [45], *Thaumetopoea pytiocampa* (Denis & Schiff.) [46] and *Ceratitis capitata* (Wied.) [47]. It has been argued, therefore, that the main drivers that shape insect gut microbial community are diet, life stage and environment [47,48]. It has been also shown that the microbial communities of insects reared on artificial diet are different from the microbiota associated with individuals from field [49].

## 5. Fungal Microbiota

Mutualistic insect-fungus associations occur among different taxa and with different strategies: bark beetles and ambrosia beetles, fungus farming ants, termites, wood wasps and gall midges are just few examples of the diversity of these associations [50,51,52]. Bark and ambrosia beetles are widely known for their strict association with fungal symbionts. Previous work has investigated the fungal community associated with bark and ambrosia beetles trapped at international harbors, revealing their association with plant pathogens and unknown fungi that they can potentially spread worldwide through the network of wood-products transportation [53]. On the other hand, there are cases of antagonistic relationships between insect and fungi, as it occurs for entomopathogenic fungi such as *Beauveria* spp. and *Metarhizium* spp. [54]. These, and other fungi, are widely employed as biocontrol agents against insect pests [55,56]. Further interactions include also peculiar multitrophic relationships, like the ability of *Metarhizium robertsii* to transfer nitrogen from infected larvae of *Galleria mellonella* (L.) to plants [57], phenomenon similarly reported for the ectomycorrhizal fungus *Laccaria bicolor* in white pine [58]. Gene horizontal transfer between the two kingdoms has been reported in aphids [59]. It has been also shown how plant-fungus interaction can help to contrast the negative effects of herbivores [60].

However, few studied focused on studying the entire fungal microbiota associated with insects, and to date collected information are restricted to Collembola [61], Lepidoptera [62], Coleoptera [63,64,65] and Diptera [66]. DNA metabarcoding allowed the analysis of the fungal microbiota of *Bactrocera oleae* (Rossi), a major pest of olive groves, revealing its association with fungal species of the genus *Colletotrichum*, agents of the devastating olive antrachnosis [67,68]. More work has been done to better understand the symbiotic relationship between ambrosia beetles and their fungal associates [63,64,69,70].

## 6. Applied Perspectives

Meta-omics pipelines are becoming increasingly accessible to a wider range of users, both in terms of cost and skills required for data analyses. These technologies have a wide range of applications, that fall beyond the study of the insects’ microbial ecology.

The study of microbial diversity associated with insects, besides to inform on biodiversity and to enhance biocontrol programs, can be source of information for the industry sector. For example, Warnecke et al. [71] used a low-throughput metagenomic analysis to study the function of hindgut microbiota in the termite *Nasutitermes ephratae* (Holmgren), revealing a wide presence of genes responsible of cellulose and xylan hydrolysis. It has been also shown that protistan communities hosted by *Coptotermes formosanus* Shiraki and *Reticulitermes flaviceps* (Oshima) are responsible to positively influence lignocellulolytic system, thus enhancing insects’ nutrition [72,73]. The use of the microbiota associated with wood-feeding beetles as source of novel enzymes, potentially useful in industrial bioprocesses, has been investigated by different studies [74,75,76,77,78,79]. Suen et al. [80] surveyed the microbial community of the leaf-cutter ants’ garden, suggesting a role of bacteria in concert with the fungus in degrading cellulose. Krishnan et al. [81] provided a review of the potential application of insect’s gut microbiome hosting bacteria with genes useful in cellulose hydrolysis, vitamin production, nitrogen fixation and many more. Indeed, the ability to isolate very specific information from the genes expressed by an entire community can be helpful in identifying candidate genes, and thus enzymes, that can improve the reliability and efficiency of industrial processes or introduce novel features [82].

Meta-omics techniques can be used to improve bio-surveillance programs, as tools to detect the arrival, origin, invasion pathways and adaptation traits of invasive species [83]. In particular, the monitoring of critical areas (e.g., ports of entry) through massive trapping is a common practice to identify the arrival of invasive insect species [84,85,86]. This process is time consuming and often requires extensive taxonomic knowledge of different systematic groups. However, the meta-omics toolkit can help to expedite this process by analyzing the entire genetic pool of single traps, and detecting not only the arrival of invasive insect species, but also likely plant pathogens [53,83,87].

Furthermore, insect-microorganism relationships could be manipulated to improve pest control, by decreasing pests’ fitness or by increasing the efficacy of pest management programs. One possible target are bacteria protecting insects from natural enemies (e.g., *Hamiltonella defensa*, *Regiella insecticola*), excluding parasitoids from the host and/or producing secondary metabolites that complete insect’s immune system [88,89]. Further research in this direction can provide striking results to enhance biocontrol programs.

DNA metabarcoding has been shown to be useful in the identification of botanical and entomological sources of honey, a valuable product subject to fraud [90]. Improvements in DNA extraction from honey, and the automatization of the procedure, together with the classification technique based on neural networks and machine learning, can lead the development of novel anti-fraud platforms.

## 7. Conclusions

We are now able to truly explore and finely investigate the insects’ microbial communities and their interaction with their hosts. Therefore, modern molecular technologies and bioinformatic tools could represent an alternative way to protect our crops, forests and ecosystems, while preserving health and environment. This is especially true, since our current climatic and social challenges are triggering radical changes in the way we approach plant protection. We are assisting to increased efforts in exploiting genomic technologies to retrieve pivotal information that can effectively be used in contrasting insect pests. Technologies and protocols are constantly improving, procedure automation is becoming increasingly common, and modern bioinformatic and machine learning workflows are helping the transition from conventional approaches. This is done by decreasing both the time-to-results and the range of skills required to correctly process samples and retrieve meaningful information. Therefore, it is now time to climb through the looking glass, search beyond the lab bench, and push our research efforts into real-world situations.
